# Proteomic insights into the biology of dopaminergic neurons

**DOI:** 10.3389/fnmol.2025.1642519

**Published:** 2025-07-30

**Authors:** Claudia Cavarischia-Rega, Karan Sharma, Julia C. Fitzgerald, Boris Macek

**Affiliations:** ^1^Department of Biology, Quantitative Proteomics, University of Tübingen, Tübingen, Germany; ^2^Department of Neurodegeneration, Hertie Institute for Clinical Brain Research, University of Tübingen, Tübingen, Germany

**Keywords:** dopaminergic neurons, iPSCs, proteomics, protein turnover, synaptosomes

## Abstract

Dopaminergic neurons, primarily located in the substantia nigra, hypothalamus, and ventral tegmental area of the brain, play crucial roles in motor control, reward, motivation, and cognition. Alterations in their function are associated with numerous neurological and psychiatric disorders, such as Parkinson’s disease, but also Schizophrenia, substance use disorders, and bipolar disorder. Recent advances in mass spectrometry-based proteomics have enabled the comprehensive profiling of protein expression, turnover, subcellular localization, and post-translational modifications at an unprecedented depth of analysis. This review summarizes the developments in proteomic approaches taken to study dopaminergic neurons. We cover findings from global and spatial proteomics studies that revealed brain region-specific protein signatures, as well as dynamic turnover of proteins and the importance of mitochondrial and synaptic proteins for the health and vulnerability of dopaminergic neurons. Combined with advanced molecular cell biology tools, such as growth in microfluidic devices, fluorescent-activated synaptosome sorting, and enzymatic proximity labeling, modern proteomics allows for investigation of synaptic and subcellular proteomes. Despite these advancements, the complexity of the human brain and its cell-specific characteristics remain a challenge. The continuing integration of advanced proteomic techniques with other -omics will eventually yield improved and mechanistic understanding of dopaminergic neurons in health and disease.

## Graphical Abstract

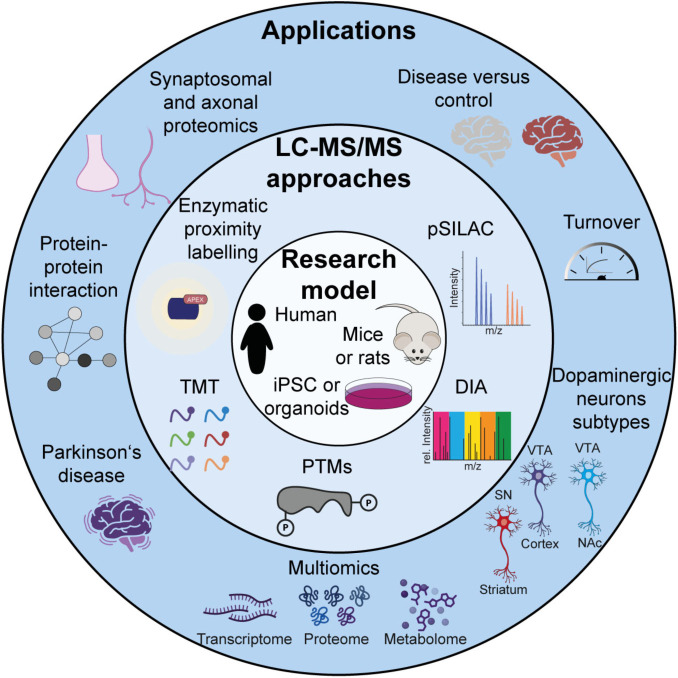

## Introduction

**FIGURE 1:**
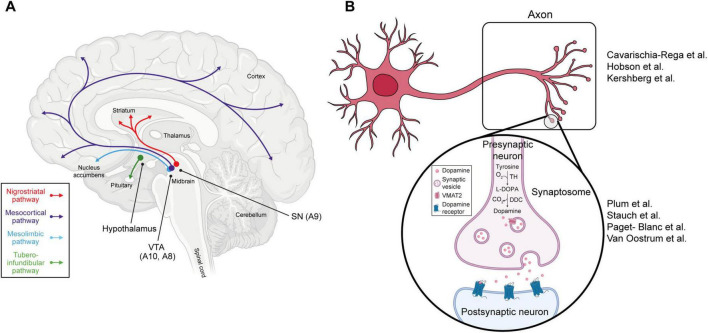
Dopamine pathways and subcellular compartments of DA neurons. **(A)** Schematic of the different brain regions where dopaminergic neurons are located. The four main dopamine pathways (nigrostriatal, mesocortical, mesolimbic, and tuberoinfundibular) are also depicted in different colors (red, purple, blue, and green respectively). **(B)** DA neuron with a zoom-in of one synapse. The key steps in the synthesis of dopamine and its release can be seen. Papers that are discussed across the review related to axonal proteomics or synaptosomal proteomics are also annotated in the figure.

Dopaminergic (DA) neurons are a specialized subset of cells in the central nervous system, distinguished by their ability to synthesize and release dopamine, a key neurotransmitter in the brain (Iversen and Iversen, 2007). These neurons are found only in certain regions of the brain, particularly the substantia nigra (SN), the ventral tegmental area (VTA) and the hypothalamus (Figure 1A). DA neurons are predominantly located in the substantia nigra pars compacta (SNpc) region of midbrain, where they are collectively referred to as the A9 group (Björklund and Dunnett, 2007). The SN is primarily composed of gray matter and has a dark coloration due to high concentrations of neuromelanin in its DA neurons. In the SN, DA neurons are involved in motor control and project into the dorsal striatum via the nigrostriatal pathway, which is essential for initiating and regulating voluntary movements (Balleine et al., 2007; Kravitz and Kreitzer, 2012). The second major population of DA neurons resides in the VTA or A10 group. In this region, DA neurons project into the nucleus accumbens (NAc) (via the mesolimbic pathway), to the prefrontal cortex (via the mesocortical pathway), and to other limbic structures (Björklund and Dunnett, 2007). These pathways mediate reward processing, motivation, learning, and cognitive functions (Floresco and Magyar, 2006; Salamone and Correa, 2012). There is another group of DA neurons called the A8 cell group, which are primarily located in the retrorubral field (Vogt Weisenhorn et al., 2016). Lastly, DA neurons are also found in the hypothalamus (group A12), mostly projecting to the infundibular structure, where they regulate mainly prolactin secretion (Grattan, 2015; Lim et al., 2025).

The intricate roles of DA neurons such as motor control, motivation, reward, and cognition, largely depend on their localization (Liss and Roeper, 2008; Wise, 2004). Alterations in the function of DA neurons can lead to several diseases (Iversen and Iversen, 2007). A well-known disease associated with DA neurons is Parkinson’s disease (PD), which is broadly characterized by progressive death of the DA neurons in the SN (Michel et al., 2016). This degeneration leads to the motor symptoms of PD, such as resting tremor, bradykinesia (slowness of movement), rigidity, and postural instability (Armstrong and Okun, 2020). While the majority of PD cases are sporadic, approximately 10–15% are familial and linked to genetic mutations (Emamzadeh and Surguchov, 2018). Autosomal dominant mutations (e.g., *SNCA*, *LRRK2*) typically result in late-onset PD, while autosomal recessive mutations (e.g., *PARK2*, *PINK1*, *PARK7*) are associated with early-onset forms. Mutations in other genes, such as *GBA1*, are associated with an increased risk (Coukos and Krainc, 2024). Apart from PD, problems in the DA signaling, particularly within the mesolimbic reward pathway, have been associated with substance abuse (e.g., opioids, alcohol, and others) (Jones et al., 2024). Alterations in DA activity and dysregulation of dopamine transporters and receptors have also been associated with bipolar disorder (BD), depression and schizophrenia (Ashok et al., 2017; Belujon and Grace, 2017).

Proteomics, the large-scale study of proteins, is a powerful tool to investigate protein networks that regulate neuronal function, synaptic plasticity, and neurological disorders (Dieterich and Kreutz, 2016; Hobson et al., 2022; van Oostrum et al., 2023; Zhao et al., 2025). In order to achieve a comprehensive understanding of the molecular basis of brain function and dysfunction and given the dynamics and complexity of the nervous system, it is crucial to study not only proteins, but also their localization, their interactions, their turnover and their post-translational modifications. Shotgun LC-MS/MS-based proteomics enables both the identification and quantification of thousands of proteins in a single experiment (Aebersold and Mann, 2016; Steen and Mann, 2004). Recent improvements in acquisition speed and sensitivity have revolutionized the field, allowing for the profiling of complete proteomes and thousands of post-translational modification sites in relatively simple experimental setups. This paper aims to provide a comprehensive overview of the recent proteomics applications in the study of DA neurons (Table 1).

**TABLE 1:** Overview on key studies included in the review.

Region				Sample type							LC-MS technique									
											Sample preparation				Quantification			Acquisition		
Stratium	idbrain - SN	Midbrain - VTA	Nucleus accumbes	iPSCs	Organoids	Mice brain	Rat brain	Human brain tissue	Genetic mutation	Key information	APEX or BioID	FASS	LCM	PTMs	SILAC	TMT or iTRAQ	LFQ	DDA	DIA	References
										PD										(Blumenreich et al., 2024)
										Turnover + multiomics										(Li et al., 2025)
										Axonal proteomics										(Hobson et al., 2022)
										PD										(Jang et al., 2023)
										Synaptosomes proteomics + PD										(Plum et al., 2020)
										Multiomics										(Zaccaria et al., 2022)
									PINK1 KO	Multiomics										(Bus et al., 2020)
										Axonal proteomics + turnover										(Cavarischia-Rega et al., 2024)
									LRRK2 G2019S	PD										(Knab et al., 2024)
									PINK1 I368N.	Multiomics										(Novak et al., 2022)
										Differences acrros DA subtypes + multiomics										(Zhao et al., 2025)
										Synaptosomes proteomics										(Kershberg et al., 2022)
										Multiomics										(Lee et al., 2020)
										Synaptic proteome										(Paget-Blanc et al., 2022)
										Synaptic proteome										(van Oostrum et al., 2023)
									PINK1 KO	Synaptosomal proteomics + PD										(Stauch et al., 2016)
										Differences acros DA subtypes + disease										(Teng et al., 2023)
										Disease + synaptosomal proteomics										(Puig et al., 2023)
									SNCA G209A	PD										(Antoniou et al., 2022)
									GBA N370S	Multiomics + PD										(Bogetofte et al., 2023)
									LRRK2 G2019S	Multiomics										(Connor-Robson et al., 2019)
										Multiomics										(Schmidt et al., 2023)
										Disease + multiomics										(Meyer et al., 2025)
										Emerging Technologies										(Wulf et al., 2022)
										Emerging Technologies										(Dutta et al., 2025)

Recent references that have been discussed are listed along with the type of sample utilized, the information they provide, and the LC-MS techniques employed.

## Research models

Historically, DA neurons were primarily studied in animal models, especially rodents, due to their ability to replicate human-like complex neural circuits and behaviors. These models also enable genetic manipulation, behavioral testing, and the ability to test drugs or compounds. However, their DA system differs significantly from that of humans, notably with a lack of neuromelanin. Post-mortem studies of human brains provided valuable insights into structural changes, but, in addition to ethical concerns, they only allow the study of late-stage diseases and lack dynamic information regarding the progression of diseases.

Another key model for researching DA neurons is the stem cells, including human embryonic stem cells (hESCs) and induced pluripotent stem cells (iPSCs). These cells can be differentiated into DA neurons and allow for disease modeling, offering a gateway toward personalized medicine. iPSCs can, for example, be derived from patients with specific genetic backgrounds and used to screen individually tailored drugs. However, they also have limitations, such as low differentiation efficiency, lack of differences across subtypes of DA neurons (Antonov and Novosadova, 2021), or the loss of epigenetic influences. An additional problem is the inability to model the complexity of all the different cell types present in regions like SN within a 2D system (Cerneckis et al., 2024). To overcome some limitations of iPSC-derived 2D neurons, brain organoids are emerging as an alternative research model. Organoids have the advantage of mimicking cell-cell interactions and cytoarchitecture better than iPSC-derived 2D neurons. However, they also have inherent limitations, such as the low reproducibility across different differentiations, a slow maturation, the absence of microglia and the absence of vasculature (Di Lullo and Kriegstein, 2017; Monzel et al., 2017; Smits and Schwamborn, 2020). Recently, approaches have been developed to generate human brain organoids with functional vascular-like systems (Cakir et al., 2019).

Although iPSC-derived dopaminergic neurons and brain organoids have advanced in vitro disease modeling, several limitations should be considered when analyzing proteomic data from these models. The heterogeneity among iPSC-derived DA neurons challenges subtype identification (Antonov and Novosadova, 2021). Many of these neurons also retain fetal-like traits, regarding their transcriptional profiles and electrical maturation, which can skew results and pose challenges for modeling age-related diseases like PD (Sison et al., 2018) or for studying human aging. Moreover, batch effects and differences across differentiations affect reproducibility. These issues highlight the importance of carefully interpreting proteomic findings from stem cell models and validating them in other systems, such as primary tissues and animal models.

## Proteomics applications in dopaminergic neuron research

### Protein expression profiling

Comprehensive protein expression profiling provides crucial information on abundance, dynamics, and basal state of proteins in DA neurons. Recent studies have mapped the transcriptome and the proteome of DA neurons, which is essential for distinguishing subtype-specific features of SNpc versus VTA DA neurons (Aguila et al., 2021; Azcorra et al., 2023; Zhao et al., 2025).

A recent study by Zhao et al. used proteomics to map the DA neuronal proteome across brain regions. It showed the proteomic landscape of NAc, SNpc, and VTA in mice at three distinct developmental stages: 7, 30, and 60 days old, corresponding to infancy, juvenile, and adult stages. They identified 443 proteins with significant temporal expression changes and reported region-specific clusters of differentially expressed proteins, revealing unique molecular features in each region at different stages. For example, proteins enriched in NAc had a role in functions such as cilium assembly, intracellular transport, and microtubule-based movement. In contrast, terms such as protein-containing complex assembly, integrin-mediated signaling pathways, and vesicle-mediated transport in synapse were enriched in the VTA region (Zhao et al., 2025).

In addition to developmental regulation, proteomic profiling has shed light on how DA neurons respond to environmental stimuli (e.g., oxidative stress, neurotoxins), pharmacological agents (e.g., L-DOPA, antipsychotics), and disease-associated genetic mutations (e.g., *LRRK2*, *SNCA*). Comparative studies across different brain regions and disease models have further enriched our understanding of the region- and context-specific molecular signatures of DA neurons (Blumenreich et al., 2024; Teng et al., 2023), which will be discussed in detail later.

**FIGURE 2:**
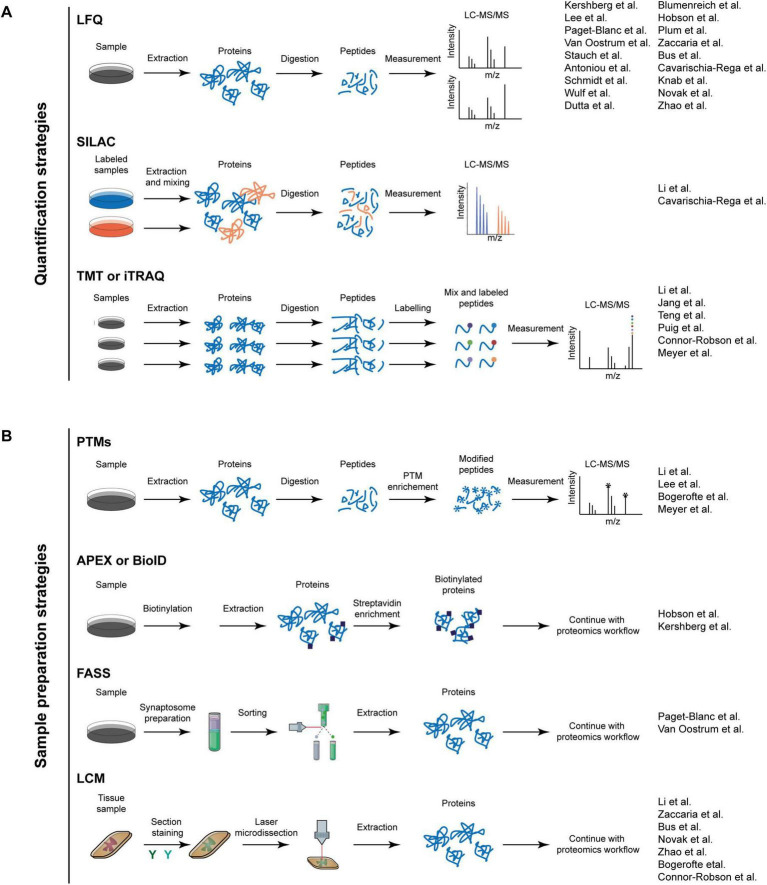
Proteomic workflows. **(A)** The workflows for quantification strategies in proteomics. The upper panel depicts a standard workflow for LFQ, wherein proteins are extracted from a sample and subsequently digested into peptides. Thereafter, the peptides are measured using LC-MS/MS. Each sample is measured independently. The middle panel shows SILAC strategy. In this approach, samples are cultivated in SILAC media, allowing the proteins to incorporate the isotopic labels during their synthesis. Following this, the proteins are extracted, mixed in a 1:1 ratio, and digested into peptides for measurement. The lower panel outlines the workflow for TMT or Isobaric Tags for Relative and Absolute Quantitation (iTRAQ) labeling. In this process, proteins are digested, and the peptides are subsequently labeled. After labeling, the peptides are mixed and measured. Quantitative information is derived from reporter ions in the MS/MS spectrum. **(B)** The strategies for sample preparation. The upper panel depicts the preparation of samples for post-translational modification (PTM) studies. Proteins are extracted and digested into peptides. Following this, a PTM enrichment step is conducted, during which unmodified peptides are removed. The samples are subsequently measured. In the upper-middle panel, the apex or BioID workflow is presented. The protein of interest, tagged with an enzyme, is activated by the addition of a reagent, resulting in the enzyme biotinylating proteins in its immediate vicinity. Cells are lysed, and the biotinylated proteins are enriched using streptavidin beads. Thereafter, proteins are digested into peptides and further analyzed with LC-MS/MS. The FASS workflow is depicted in the lower-middle panel. Initially, synaptosomes are enriched and their subpopulations are sorted using fluorescent tagging of synaptic markers. Subsequently, proteins are digested and measured. In the lower panel, the LCM workflow is illustrated, where a tissue sample is stained with various antibodies, and a specific region is microdissected and isolated using a laser. This is followed by a standard proteomic workflow. The papers discussed in the review are indicated adjacent to the workflow utilized.

Although neurons are post-mitotic, their proteins are in a constant state of synthesis and degradation, a dynamic process called protein turnover, defined by protein half-life. One widely used method for studying protein turnover is stable isotope labeling by amino acids in cell culture (SILAC), combined with mass spectrometry (MS)-based shotgun proteomics (Ong et al., 2002; Figure 2A). In this approach, cells are first grown in media containing “light” amino acids and switched to media containing “heavy” isotopic versions. As proteins are being newly synthesized, they will incorporate the heavy amino acids, which will increase the protein mass. This mass difference can be detected using mass spectrometry and increase of heavy peak intensities can be monitored over time. This technique, termed “dynamic” or “pulsed” SILAC, provides valuable insights into protein synthesis rates, degradation, and overall proteome dynamics (Doherty et al., 2009; Li, 2010).

Li et al. applied in vivo dynamic SILAC labeling in mice, using labeled food, to study protein turnover across 16 tissues and brain regions, including the striatum and the SN. Notably, proteins of the mitochondrial respiratory chain complex I exhibited both high abundance and long half-lives in the brain compared to other tissues and other mitochondrial proteins, emphasizing the importance of oxidative phosphorylation in the brain. Their study also revealed that phosphorylation can influence protein stability and turnover (Li et al., 2025).

Cavarischia-Rega et al. used pSILAC to measure the half-lives of approximately 4,300 proteins in iPSCs derived human DA neurons, reporting a median half-life of 97 h. Interestingly, mitochondrial ribosome proteins had significantly shorter half-lives than the cytosolic ribosomes. Moreover, large and small subunits of the cytosolic ribosome had significantly different half-lives. Additionally, long-lived proteins were enriched in pathways related to cytoskeletal regulation by Rho GTPases, pyruvate metabolism, and de novo purine and cholesterol biosynthesis. In contrast, synaptic vesicle trafficking, Wnt signaling, and Alzheimer’s disease-amyloid and presenilin pathways were overrepresented among the most short-lived proteins (Cavarischia-Rega et al., 2024).

Proteomic profiling helps in the characterization of the differences between subtypes of DA neurons and their response to diverse stimuli, while studying protein turnover allows for a dynamic picture of the proteome in DA neurons. However, this information is only present at the cell level in a general population.

### Advances in spatial and subcellular proteomics

Spatial proteomics is an emerging field that is focused on identifying the tissue-specific and subcellular localization of proteins and understanding how this spatial localization regulates signaling and function. Synapses are highly specialized key compartments of the neurons, which explains the high relevance of their study (Figure 1B). One notable study by Plum et al. investigated the synaptosomal proteome from postmortem human SN samples of PD patients and healthy controls. They identified 362 proteins within the synaptosomal core proteome, out of which 14 proteins were underrepresented in PD, several of which are related to mitochondrial translation. This highlights mitochondrial dysfunction at the synaptic level in PD (Plum et al., 2020).

Recently, Cavarischia-Rega et al. analyzed the enriched axonal proteins in iPSCs derived human DA neurons with the help two-well microfluidic devices, identifying 127 axon-enriched proteins. They also performed differential dynamic SILAC labeling to monitor protein trafficking, which resulted in the detection of translocation of 269 proteins between axons and the soma in the time frame of the analysis (120 h). Importantly, they provided evidence for local synthesis of 154 proteins in the axon and their retrograde transport to the soma, including several proteins involved in RNA biology, such as adenosine deaminase ADAR and RNA helicase DHX30 (Cavarischia-Rega et al., 2024).

Further emphasizing the role of mitochondria, Stauch et al. studied synaptic mitochondria in the striatum of 3-month-old PINK1 knockout and wild-type rat brains. They identified 811 proteins, of which 69 were differentially expressed. The most enriched protein functions were related to the electron transport chain and oxidation-reduction (Stauch et al., 2016). These findings again underline the mitochondrial signature in DA neuron synapses, in particular the respiratory chain. Cavarischia-Rega et al. analyzed the turnover rate of protein subunits of the respiratory chain in dopaminergic neurons and compared this to similar data from human cancer cells (Morgenstern et al., 2021), which revealed that the turnover of mitochondrially encoded subunits remains stable, whereas nuclear encoded subunits differ in their turnover rate between these two very different cell types. Such a comparison of freely available proteomic datasets for specific cell types is a useful resource and offers mechanistic insight for future research.

An important development to study synapses was fluorescence-activated synaptosomes sorting (FASS), which relies on fluorescent tagging of synaptic markers followed by fluorescence-activated cell sorting, enabling high-purity isolation of specific synaptic populations (Luquet et al., 2017; Figure 2B). This technique has been utilized in several proteomics studies to elucidate the complexity of DA synapses. Paget-Blanc et al. combined FASS with label-free quantitative (LFQ) data-dependent acquisition (DDA) proteomics to look into the synapses of the striatum in midbrain mice, identifying 57 highly enriched proteins at the DA synapses. These proteins were related to release sites, vesicles, mitochondria, and cytoskeletal elements. They also described “dopamine hub synapses,” showing that DA synapses associate with cholinergic (14%), GABAergic (26%), and glutamatergic (42%) partners, highlighting the complex microenvironment of DA terminals and underscores the relevance of using organoids over 2D cultures (Paget-Blanc et al., 2022). Van Oostrum et al. also used FASS to investigate synaptosomal differences across different neuronal types in mice, including DA neurons. They focused on the synaptic proteome of striatal DA terminals, where they described 267 proteins significantly enriched at DA terminals. They also revealed novel synapse-enriched proteins specifically for this neuron type, such as MAPK3, ATP6V1G1 or KIF5C. Remarkably, OXR1 (Oxidation resistance protein 1), a protein involved in oxidative stress protection, was absent in DA synaptosomes but present in others, which could potentially explain the vulnerability of DA neurons to oxidative stress (van Oostrum et al., 2023).

Another major advancement in proteomics was the use of enzymatic proximity labeling, which allows the study of protein-protein interactions and subcellular localization. Briefly, the protein of interest or “bait” is tagged with an enzyme, typically a biotin ligase, which, upon the addition of a reagent, gets activated. Upon activation, the enzyme biotinylates proteins in its instant proximity, typically within a 10–20 nm radius. Cells are lysed, and the biotinylated proteins are enriched with streptavidin beads and further analyzed with LC-MS/MS (Figure 2B). The two most commonly used enzymes are the mutant biotin ligase *(*BioID) (Roux et al., 2012) and the ascorbate peroxidase (APEX) (Rhee et al., 2013). BioID is slower (acts within hours) and has a labeling radius of ∼10 nm, while APEX is much faster (acts within minutes) and has a radius of ∼20 nm (Xu et al., 2021).

Using APEX2, Hobson et al. conducted a comprehensive mapping of somatodendritic and axonal proteomes of DA neurons from mouse midbrain slice. Their analysis revealed that 373 proteins were unique to the ventral midbrain (VM) and 708 to the striatum. In the somatodendritic samples, protein functions associated with protein synthesis and degradation machinery were enriched. Conversely, the axonal/striatum samples were enriched for autophagy, endocytosis, and glycolysis of transport. They also described the unexpected axonal localization of the potassium channels Kv4.3 and GIRK2, which were usually thought to only be present in the soma (Hobson et al., 2022). Finally, Kershberg et al. used BioID to investigate the proteomic composition of dopamine release sites in the mouse striatum. They identified 527 enriched proteins, which were mainly involved in secretory machinery, calcium regulation, and synaptic vesicle function (Kershberg et al., 2022). Notably, their study had a 65% overlap of axonal proteins with those identified by Hobson et al. (2022). Taken together, spatial proteomics has given new insights into the dynamic localization and interaction of DA neurons’ proteins.

### Post-translational modifications in DA neurons

Post-translational modifications (PTMs), such as phosphorylation, acetylation, and ubiquitination, play a crucial role in cell signaling. PTMs critically modulate protein function and are often perturbed in disease (Luo et al., 2023; Wu et al., 2023). Phosphorylation can control receptor activation, ion channel function, and neurotransmitter release (Comenencia-Ortiz et al., 2014; Foster and Vaughan, 2017). However, this field is quite understudied, and there are only a few studies in DA neurons. One major limitation of PTMs study is the need for an enrichment step that requires a relatively high initial protein amount (Figure 2B). The previously discussed research by Li et al. explored the phosphoproteome and the influence of specific phosphorylation events on the protein lifetime in the mouse brain (Li et al., 2025). Another example is the study by Meyer et al., which investigated phosphoproteomic profiles in brain organoids derived from patients with BD (Meyer et al., 2025).

Bogerofte et al performed a comprehensive PTM analysis (phosphorylation, sialylation, and cysteine modifications) on iPSCs-derived neurons from four patients carrying a GBA-N370S mutation and four control iPSCs lines. In total, they identified 7,988 phosphorylation sites (on 3,092 proteins), 2,862 N-linked glycosites (on 1,055 proteins), and 11,148 reversible cysteine modifications (on 4,456 proteins). Among the phospho-proteins, functions related to RNA biology, such as RNA splicing or regulation of mRNA metabolic process were enriched whereas for the reversible cysteine-modified PTMs, RNA localization and ribonucleoprotein complex biogenesis were some of the significantly regulated protein functions (Bogetofte et al., 2023).

Lee et al. investigated the role of O-GlcNAcylation, a dynamic glycosylation modification, in the mouse brain. They identified 926 proteins in the SNpc-VTA region and 919 in the striatum. Proteins linked to pathways related to neuronal and synaptic structure and function, including dopamine receptor-mediated signaling, show abundant O-GlcNAcylation. Interestingly, the known marker proteins Tyrosine Hydroxylase (TH) and Dopamine Transporter (DAT) were found to be potentially O-GlcNAcylated (Lee et al., 2020). In summary, these studies show the relevance of PTMs, as they are shown to influence the regulation of multiple pathways including, synaptic function, RNA-related processes, and also protein turnover.

## Applications of proteomics in disease

Quantitative proteomics, particularly using tandem mass tag (TMT) labeling, has become a common and powerful approach tool for studying protein alterations in disease contexts. This method uses isobaric chemical tags that are covalently attached to peptides, allowing both identification and relative quantification of proteins across complex experimental conditions (Thompson et al., 2003; Figure 2A). It has a high multiplexing capacity, allowing the comparison of protein expression across multiple biological samples simultaneously, highlighting its relevance in the study of human brain tissue from patients and healthy controls.

### Parkinson’s disease

Proteomics has highlighted the involvement of mitochondrial impairment in PD, where DA neurons have a critical role. Functional proteomics of samples with altered expression of LRRK2, GBA1, or α-synuclein (αSyn) has revealed how these proteins disrupt cellular pathways when mutated or misfolded. For example, Blumenreich et al. performed proteomics on five brain regions, including the SN and the striatum, from control, idiopathic PD (IPD), and PD with GBA1 mutation (PD-GBA) patients. This allowed the comparison of protein changes across different brain regions within the same patients. When comparing the IPD and PD-GBA, only two proteins were significantly altered in most brain regions: GCase and ADl1. Mitochondrial oxidative phosphorylation was impaired in PD brains, but more so in PD-GBA cases, reinforcing the link between mitochondrial dysfunction and PD (Blumenreich et al., 2024).

In a proteomic analysis of human SN from 15 PD and 15 healthy control patients, Jang et al. used TMT to identify over 10,000 proteins. Among these, 1,140 were differentially expressed in the SN. They discovered downregulation of mitoribosome proteins in PD, while RNA splicing and complement proteins were upregulated (Jang et al., 2023).

As previously mentioned, the use of patient-derived iPSCs opens the possibility for discovering new therapeutics. This was the case in the study performed by Antoniou et al., where iPSCs derived from a patient with the p.A53T αSyn mutation (G209A in the *SNCA* gene) were used to study the effect of the multi-kinase inhibitor BX795. To identify compounds with possible neuroprotective properties, the author’s performed a screening of 273 small-molecule kinase inhibitors. Only BX795, an aminopyrimidine compound, showed a consistent dose-response effect. It significantly restored proteins associated with RNA metabolism, protein synthesis, modification, and clearance, and stress response (Antoniou et al., 2022).

Knab et al. analyzed both the whole proteome and the extracellular vesicles of iPSCs from a patient carrying the LRRK2 G2019S mutation and an isogenic gene-corrected control. In the extracellular vesicles, they detected 484 significantly differentially regulated proteins, whereas in cell lysates, they detected 1,833 proteins. In the 123 proteins detected in both datasets, they reported 10 potentially novel biomarkers. Some examples were SHH (sonic hedgehog signaling molecule), RHOA (Ras homolog family member A) and CD44, which were upregulated, or GALC (galactosylceramidase), ANXA1 (annexin A1) and AGO1 (argonaute RISC component 1), which were downregulated (Knab et al., 2024). These findings illustrate the power of combining proteomics with disease-relevant cell models for biomarker discovery.

### Neuropsychiatric disorders

Beyond PD, proteomics has been employed to uncover the molecular underpinnings of other neuropsychiatric conditions. In alcohol use disorder (AUD), Teng et al. used TMT to analyze multiple different brain regions, including the NAc and VTA, from the brains of 16 male AUD patients and 11 healthy individuals. They found 34 proteins to be differently expressed in the NAc, with lysine degradation and urea cycle pathways enriched. In the VTA, there were 29 proteins differently expressed, with acute phase response signaling being enriched, and six proteins associated with this pathway were elevated in AUD (Teng et al., 2023).

Similarly, Puig et al. studied bulk tissue and synaptosome preparations from the dorsolateral prefrontal cortex (DLPFC) and the NAc from human brains with opioid use disorder (OUD). In the NAc, they identified four upregulated and 29 downregulated proteins in the total proteome and 17 upregulated and 39 downregulated in the synaptosomes. Overall, they showed significant alterations in circadian rhythm, GABAergic, and glutamatergic synaptic processes in both regions of the OUD patients. When looking specifically into the regulated pathways in the NAc synaptosomes, they found mitochondrial and endoplasmic reticulum functions, along with second messenger signaling cascades, to be enriched. They also investigated rhythmic proteins (proteins that fluctuate in sync with circadian rhythms) and their changes between unaffected and OUD subjects in synaptosomes. In OUD, 23 proteins lost rhythmicity in the NAc, while seven did so in the DLPFC. Conversely, 11 proteins gained rhythmicity in DLPFC and none on NAc in the OUD samples (Puig et al., 2023).

As mentioned previously, Meyer et al. developed midbrain organoids derived from iPSCs from patients with BD and healthy controls, with a population of 60-70% of DA neurons. Using TMT multiplexing, they found 234 significantly differentially expressed proteins in BD organoids compared to the controls. Upregulated proteins had enriched functions such as carboxylic acid binding, lysophospholipase activity, and spine morphogenesis. On the other hand, downregulated proteins were mainly related to signaling pathways, including adenylate cyclase activity, NADPH binding, and protein autophosphorylation. They also performed phosphoproteomics, where they identified 196 differentially phosphorylated proteins. Interestingly, synapse and postsynaptic specialization processes, RNA-silencing, ribonucleoprotein granules, and P-bodies processes were observed among the cluster of hypo-phosphorylated proteins. In contrast, hyper-phosphorylated proteins were involved in phosphatidylinositol (PI) signaling system, a known target of the mood-stabilizing drug lithium. These findings show the potential link between molecular alterations and therapeutic mechanisms (Meyer et al., 2025).

Across neurodegenerative and psychiatric disorders, proteomics (especially when coupled with models like patient-derived iPSCs and advanced technologies such as TMT labeling) has proven to be particularly useful in revealing mechanistic insights into disease biology, identifying biomarkers, and facilitating therapeutic discovery. Shared proteomic signatures are emerging across neurodegenerative and psychiatric disorders. Mitochondrial and synaptic dysfunctions were observed not only in PD but also in AUD and OUD, indicating common vulnerability pathways. Interestingly, defects of mitochondrial function and synaptic connectivity are associated with Schizophrenia, a condition affecting both dopaminergic and cortical pathways (Breitmeyer et al., 2023).

Nevertheless, each condition has distinct changes; for example, PD uniquely involves dysregulated lysosomal function, alpha-synuclein pathology, and dopaminergic neuron degeneration, which are not typical features of AUD, BD, or OUD. Furthermore, the primary subtype of DA neuron affected varies, with PD mainly affecting SNpc DA neurons, while VTA DA neurons are more involved in psychiatric disorders. These findings suggest some overlap in DA-related pathophysiology but highlight the need for disorder-specific proteomic analysis. In this regard, further cell type specific and disease specific (genetic models, patient derived or treatment) studies will offer a comprehensive picture of the proteomic landscape of the human brain in health and disease.

## Integration with other -omics

Multi-omics approaches enable the construction of comprehensive molecular maps of DA neurons. Numerous studies on DA neurons have been done on the transcriptomics level (Birtele et al., 2022; Chung et al., 2005; Fiorenzano et al., 2024; Jerber et al., 2021; Kamath et al., 2022; Krauskopf et al., 2022; Mandel et al., 2007). Despite the overall insight provided by these studies, it is important to note that the mRNA levels do not always correlate with proteome levels (Schwanhäusser et al., 2011). This difference has been shown to be even more pronounced for axonal proteins (Moritz et al., 2019).

Some recent studies are starting to integrate different -omics analyses. For example, Zhao et al., performed SMART-seq2 transcriptomics and data-independent acquisition (DIA) proteomics of different regions of the mouse brain (NAc, SNpc, and VTA) at different stages of development. The overall correlation between transcriptome and proteome was moderate and consistent with other studies. This highlights the complexity of transcriptional and translational regulation. However, ALDH1A1 (Aldehyde dehydrogenase 1A1) showed good consistency between transcription and translation. Both the protein and mRNA levels had a progressive increase across all regions, mainly in the VTA, suggesting its potential as a marker for DA neuron maturation (Zhao et al., 2025). Using iPSCs derived from fibroblasts from seven sporadic PD patients and five age- and sex-matched controls, Schmidt et al. performed an integrated transcriptomics, proteomics, and metabolomics study. They found that the TCA cycle, specifically the α-ketoglutarate dehydrogenase complex, is a bottleneck in sporadic PD metabolism (Schmidt et al., 2023). Bus et al. used homozygous PINK1 KO iPSCs-derived midbrain specific neurons to perform metabolomics, transcriptomics and proteomics. Across all the multi-omics experiments, nucleotide and amino acid metabolism were highlighted in PINK1 KO hDANs. In particular, protein functions related to amide metabolism were enriched when transcriptomic and proteomic changes were integrated (Bus et al., 2020). Connor-Robson et al. integrated transcriptomics and proteomics in iPSCs-derived DA neurons carrying the *LRRK2-G2019S* mutation (Connor-Robson et al., 2019), whereas Novak et al. used midbrain DA iPSCs carrying the I368N-PINK1 mutation and applied single-cell RNA-seq and proteomics (Novak et al., 2022).

Despite the relevance of proteomics, it is important to integrate the data with different omics, since they provide different layers of information. This is especially relevant considering that the correlation between mRNA and protein levels is quite low for axonal proteins.

## Emerging technologies in proteomics and future applications

The future of DA neuron proteomics lies in improving sensitivity and spatial resolution. One technique that can achieve this is laser capture microdissection (LCM), used to isolate specific cells or microscopic regions of tissue from heterogeneous samples. A focused laser beam is used to cut the area of interest, which later can be used to perform proteomics (Espina et al., 2006; Figure 2B).

Wulf et al. used LCM for isolated NMGs (neuromelanin granules) and surrounding SN tissue from control patients. Proteomic analysis revealed enrichment of lysosomal proteins in NMGs, confirming the previously described link between NMGs and lysosomes. TH was also highly abundant in NMGs (Wulf et al., 2022). Zaccaria et al. used LCM to study DA from SNpc from five PD patients and five control subjects, followed by RNAseq and proteomics. They observed differential expression of 52 genes and 33 proteins. As in previous reports, the correlation between RNA and protein expression was low. Notably, CST3 (cystatin-C) upregulation in PD samples was the only consistent finding across the transcriptomic and proteomic datasets. Some genes, like *S100 B* and *MAOA*, were upregulated at the RNA level but showed no changes at the proteome level, and some others (e.g., *MT1H*, *CXCR4)* were not even identified in the proteomic data despite their upregulation at the RNA level. Interestingly, ALDH1A1 protein levels were decreased in PD samples (Zaccaria et al., 2022), contrary the previously noted correlation in the Zhao et al. study (Zhao et al., 2025).

A promising emerging field is single-cell proteomics, which is advancing rapidly (Guo et al., 2025). This approach will provide a key understanding of DA neuron heterogeneity, including their differential susceptibility to disease. Single cell proteomics could be applied to characterize neuronal subtypes from different brain regions as well as non-neuronal cells like astrocytes. Dutta et al. employed spatial single cell-proteomics through immunostaining- LCM on cortex and SN from mouse brain. They compared ventral SNpc to dorsal SN neurons, and identified ALDH1A1 as one of the upregulated hits in the ventral SN neurons (Dutta et al., 2025). Zheng et al. recently introduce mipDVP, a new cutting-edge imaging proteomics method combining multiplexed imaging, single-cell laser microdissection, and mass spectrometry (Zheng et al., 2025). This tool could be directly applied into new research to look into the proteomes of the different subpopulation of DA neurons allowing to study their distinct proteomic signatures and vulnerabilities. Sabatier et al. measure the protein turnover and abundance from single cells, in particular from differentiated cells (Sabatier et al., 2025). Although this was not specifically done on DA neurons, this technique could also be applied to investigate about differences in turnover of the subtypes of DA neurons, perhaps in combination with FASS.

Beyond cell and tissue specificity, proteomics also holds promise for biomarker discovery and disease prediction. Artificial intelligence (AI) and machine learning algorithms could be applied to identify novel patterns and predictive biomarkers from complex proteomic datasets. When integrated with multi-omics data, such as transcriptomics, metabolomics, and epigenomics, AI-driven models could enhance precision medicine approaches by offering personalized therapeutic targets. The use of AI for predictive biomarker discovery is already starting to be used in the field of oncology (Alum, 2025; Prelaj et al., 2024) and even on neurodegenerative diseases (Kitaoka et al., 2025).

While emerging techniques like spatial and single-cell proteomics offer unprecedented resolution, they are not without challenges. Low proteome coverage remains a significant limitation, especially in single-cell studies where total protein yield is in the picogram range (a single cell typically contains from 50 to 500 pg). Due to this, quantitation of the data can be noisy and less reproducible. Additionally, complex equipment is required for cell isolation and sample preparation (Rosenberger et al., 2023). High data sparsity, batch effects, and technical variability also hinder reproducibility. Methods like LCM and proximity labeling also require specialized equipment and often suffer from low throughput. LCM also requires tissue processing and microdissection which can cause protein degradation or modification, reducing data quality. Addressing these limitations is important for the broad adoption of next-generation proteomic technologies.

## Conclusion

Beyond their descriptive power, proteomic studies have provided critical mechanistic insights into DA neuron biology. For instance, spatial proteomic approaches have revealed an unexpected subcellular distribution of canonical and non-canonical synaptic proteins (Cavarischia-Rega et al., 2024; Hobson et al., 2022), such as the axonal localization for potassium channels like Kv4.3 and GIRK2, suggesting previously unappreciated roles in presynaptic excitability and dopamine release dynamics (Hobson et al., 2022). Moreover, protein turnover studies have highlighted the dynamic nature of vesicle trafficking and neurotransmission machinery. Synaptic vesicle trafficking proteins exhibit some of the shortest half-lives in iPSC-derived DA neurons (Cavarischia-Rega et al., 2024), indicating tight regulation and rapid remodeling of dopamine synaptic release mechanisms. The use of proteomics has also unveiled key protein–protein interactions at DA synapses, identifying machinery enriched in calcium regulation and vesicular exocytosis, which are critical for dopamine release and reuptake (Hobson et al., 2022; Kershberg et al., 2022; Paget-Blanc et al., 2022; van Oostrum et al., 2023). Mitochondrial proteomics has consistently reported the downregulation of mito-ribosome and oxidative phosphorylation components across multiple studies (Blumenreich et al., 2024; Jang et al., 2023; Li et al., 2025; Plum et al., 2020; Stauch et al., 2016), implicating that impaired mitochondrial bioenergetics directly contributes to DA neuron vulnerability. Additionally, the absence of antioxidant proteins like OXR1 from DA synapses (van Oostrum et al., 2023) suggested a mechanistic basis for selective oxidative stress sensitivity. Finally, proteomic profiling of disease models has revealed alterations in different pathways, which provide a biochemical explanation for observed phenotypes in PD models, such as impaired neurite outgrowth, vesicle recycling, and axonal transport (Bogetofte et al., 2023; Knab et al., 2024).

Overall, continuous advancements in proteomics technologies provide a key opening for future research on DA neurons. By directly measuring protein abundance and modification states, proteomics offers a functional readout of cellular processes that may not always be reflected at the transcriptomic level. Another significant advantage is the high-throughput and unbiased nature of mass spectrometry-based proteomics, which can identify and quantify thousands of proteins in a single experiment.

However, intrinsic characteristics of DA neurons, such as their heterogeneity, sparse distribution, and deep localization in the brain, present technical challenges. DA neurons are quite heterogeneous and are localized in deep brain structures, impeding direct access for experimental interventions, making it challenging to obtain sufficient quantities for analysis without contamination from other cell types. Even if enough material is obtained, the wide dynamic range of protein abundance can mask low-copy proteins. Furthermore, many PTMs are transient, sub-stoichiometric, and biochemically fragile, increasing the difficulty of their study. Also, proteins present very different half-lives, increasing the complexity of any kind of dynamic study. Finally, comprehensive proteomic studies still require expensive instrumentation, special expertise, and considerable bioinformatics support, which impedes their accessibility. Single cell and spatial proteomics, with better mass analyzers and techniques such as mipDVP, will enable a more comprehensive study of the different DA subtypes and their synaptic microenvironments. As proteomic technologies continue to evolve, their application across disease contexts will not only deepen our molecular insights but also pave the way for precision medicine in neurology and psychiatry.

In conclusion, continued technical innovation, open access data sharing, and interdisciplinary integration will be essential for translating new insights into mechanistic understanding of dopaminergic neurons and, ultimately, therapeutic benefit.

## References

[B1] Aebersold R. Mann M. (2016). Mass-spectrometric exploration of proteome structure and function. *Nature* 537 347–355. 10.1038/nature19949 27629641

[B2] Aguila J. Cheng S. Kee N. Cao M. Wang M. Deng Q. (2021). Spatial RNA sequencing identifies robust markers of vulnerable and resistant human midbrain dopamine neurons and their expression in Parkinson’s disease. *Front. Mol. Neurosci.* 14:699562. 10.3389/fnmol.2021.699562 34305528 PMC8297217

[B3] Alum E. U. (2025). AI-driven biomarker discovery: Enhancing precision in cancer diagnosis and prognosis. *Discov. Oncol.* 16:313. 10.1007/s12672-025-02064-7 40082367 PMC11906928

[B4] Antoniou N. Prodromidou K. Kouroupi G. Boumpoureka I. Samiotaki M. Panayotou G. (2022). High content screening and proteomic analysis identify a kinase inhibitor that rescues pathological phenotypes in a patient-derived model of Parkinson’s disease. *NPJ Parkinsons Dis.* 8:15. 10.1038/s41531-022-00278-y 35149677 PMC8837749

[B5] Antonov S. Novosadova E. (2021). Current state-of-the-art and unresolved problems in using human induced pluripotent stem cell-derived dopamine neurons for Parkinson’s disease drug development. *Int. J. Mol. Sci.* 22:3381. 10.3390/ijms22073381 33806103 PMC8037675

[B6] Armstrong M. Okun M. (2020). Diagnosis and treatment of parkinson disease: A review. *JAMA* 323 548–560. 10.1001/jama.2019.22360 32044947

[B7] Ashok A. Marques T. Jauhar S. Nour M. Goodwin G. Young A. (2017). The dopamine hypothesis of bipolar affective disorder: The state of the art and implications for treatment. *Mol. Psychiatry* 22 666–679. 10.1038/mp.2017.16 28289283 PMC5401767

[B8] Azcorra M. Gaertner Z. Davidson C. He Q. Kim H. Nagappan S. (2023). Unique functional responses differentially map onto genetic subtypes of dopamine neurons. *Nat. Neurosci.* 26 1762–1774. 10.1038/s41593-023-01401-9 37537242 PMC10545540

[B9] Balleine B. Delgado M. Hikosaka O. (2007). The role of the dorsal striatum in reward and decision-making. *J. Neurosci.* 27 8161–8165. 10.1523/JNEUROSCI.1554-07.2007 17670959 PMC6673072

[B10] Belujon P. Grace A. (2017). Dopamine system dysregulation in major depressive disorders. *Int. J. Neuropsychopharmacol.* 20 1036–1046. 10.1093/ijnp/pyx056 29106542 PMC5716179

[B11] Birtele M. Storm P. Sharma Y. Kajtez J. Wahlestedt J. Sozzi E. (2022). Single-cell transcriptional and functional analysis of dopaminergic neurons in organoid-like cultures derived from human fetal midbrain. *Development* 149:dev200504. 10.1242/dev.200504 36305490 PMC10114107

[B12] Björklund A. Dunnett S. (2007). Dopamine neuron systems in the brain: An update. *Trends Neurosci.* 30 194–202. 10.1016/j.tins.2007.03.006 17408759

[B13] Blumenreich S. Nehushtan T. Kupervaser M. Shalit T. Gabashvili A. Joseph T. (2024). Large-scale proteomics analysis of five brain regions from Parkinson’s disease patients with a GBA1 mutation. *NPJ Parkinsons Dis.* 10:33. 10.1038/s41531-024-00645-x 38331996 PMC10853186

[B14] Bogetofte H. Ryan B. Jensen P. Schmidt S. Vergoossen D. Barnkob M. (2023). Post-translational proteomics platform identifies neurite outgrowth impairments in Parkinson’s disease GBA-N370S dopamine neurons. *Cell. Rep.* 42:112180. 10.1016/j.celrep.2023.112180 36870058 PMC7617855

[B15] Breitmeyer R. Vogel S. Heider J. Hartmann S. Wüst R. Keller A. (2023). Regulation of synaptic connectivity in schizophrenia spectrum by mutual neuron-microglia interaction. *Commun. Biol.* 6:472. 10.1038/s42003-023-04852-9 37117634 PMC10147621

[B16] Bus C. Zizmare L. Feldkaemper M. Geisler S. Zarani M. Schaedler A. (2020). Human dopaminergic neurons lacking PINK1 exhibit disrupted dopamine metabolism related to vitamin B6 Co-factors. *iScience* 23:101797. 10.1016/j.isci.2020.101797 33299968 PMC7702004

[B17] Cakir B. Xiang Y. Tanaka Y. Kural M. Parent M. Kang Y. (2019). Engineering of human brain organoids with a functional vascular-like system. *Nat. Methods* 16 1169–1175. 10.1038/s41592-019-0586-5 31591580 PMC6918722

[B18] Cavarischia-Rega C. Sharma K. Fitzgerald J. Macek B. (2024). Proteome dynamics in iPSC-derived human dopaminergic neurons. *Mol. Cell. Proteomics* 23:100838. 10.1016/j.mcpro.2024.100838 39251023 PMC11474371

[B19] Cerneckis J. Cai H. Shi Y. (2024). Induced pluripotent stem cells (iPSCs): Molecular mechanisms of induction and applications. *Signal. Transduct. Target Ther.* 9:112. 10.1038/s41392-024-01809-0 38670977 PMC11053163

[B20] Chung C. Seo H. Sonntag K. Brooks A. Lin L. Isacson O. (2005). Cell type-specific gene expression of midbrain dopaminergic neurons reveals molecules involved in their vulnerability and protection. *Hum. Mol. Genet.* 14 1709–1725. 10.1093/hmg/ddi178 15888489 PMC2674782

[B21] Comenencia-Ortiz E. Moss S. Davies P. (2014). Phosphorylation of GABAA receptors influences receptor trafficking and neurosteroid actions. *Psychopharmacology* 231 3453–3465. 10.1007/s00213-014-3617-z 24847959 PMC4135009

[B22] Connor-Robson N. Booth H. Martin J. Gao B. Li K. Doig N. (2019). An integrated transcriptomics and proteomics analysis reveals functional endocytic dysregulation caused by mutations in LRRK2. *Neurobiol. Dis.* 127 512–526. 10.1016/j.nbd.2019.04.005 30954703 PMC6597903

[B23] Coukos R. Krainc D. (2024). Key genes and convergent pathogenic mechanisms in Parkinson disease. *Nat. Rev. Neurosci.* 25 393–413. 10.1038/s41583-024-00812-2 38600347

[B24] Di Lullo E. Kriegstein A. (2017). The use of brain organoids to investigate neural development and disease. *Nat. Rev. Neurosci.* 18 573–584. 10.1038/nrn.2017.107 28878372 PMC5667942

[B25] Dieterich D. Kreutz M. (2016). Proteomics of the synapse–A quantitative approach to neuronal plasticity. *Mol. Cell. Proteomics* 15 368–381. 10.1074/mcp.R115.051482 26307175 PMC4739661

[B26] Doherty M. Hammond D. Clague M. Gaskell S. Beynon R. (2009). Turnover of the human proteome: Determination of protein intracellular stability by dynamic SILAC. *J. Proteome Res.* 8 104–112. 10.1021/pr800641v 18954100

[B27] Dutta S. Pang M. Coughlin G. Gudavalli S. Roukes M. Chou T. (2025). Molecularly-guided spatial proteomics captures single-cell identity and heterogeneity of the nervous system. *bioRxiv* [Preprint] 10.1101/2025.02.10.637505 39990460 PMC11844393

[B28] Emamzadeh F. Surguchov A. (2018). Parkinson’s disease: Biomarkers, treatment, and risk factors. *Front. Neurosci.* 12:612. 10.3389/fnins.2018.00612 30214392 PMC6125353

[B29] Espina V. Milia J. Wu G. Cowherd S. Liotta L. (2006). Laser capture microdissection. *Methods Mol. Biol.* 319 213–229. 10.1007/978-1-59259-993-6_10 16719357

[B30] Fiorenzano A. Storm P. Sozzi E. Bruzelius A. Corsi S. Kajtez J. (2024). TARGET-seq: Linking single-cell transcriptomics of human dopaminergic neurons with their target specificity. *Proc. Natl. Acad. Sci. U S A.* 121:e2410331121. 10.1073/pnas.2410331121 39541349 PMC11588066

[B31] Floresco S. Magyar O. (2006). Mesocortical dopamine modulation of executive functions: Beyond working memory. *Psychopharmacology (Berl)* 188 567–585. 10.1007/s00213-006-0404-5 16670842

[B32] Foster J. Vaughan R. (2017). Phosphorylation mechanisms in dopamine transporter regulation. *J. Chem. Neuroanat.* 83-84 10–18. 10.1016/j.jchemneu.2016.10.004 27836487 PMC6705611

[B33] Grattan D. (2015). 60 years of neuroendocrinology: The hypothalamo-prolactin axis. *J. Endocrinol.* 226 T101–T122. 10.1530/JOE-15-0213 26101377 PMC4515538

[B34] Guo T. Steen J. Mann M. (2025). Mass-spectrometry-based proteomics: From single cells to clinical applications. *Nature* 638 901–911. 10.1038/s41586-025-08584-0 40011722

[B35] Hobson B. Choi S. Mosharov E. Soni R. Sulzer D. Sims P. (2022). Subcellular proteomics of dopamine neurons in the mouse brain. *Elife* 11:e70921. 10.7554/eLife.70921 35098924 PMC8860448

[B36] Iversen S. Iversen L. (2007). Dopamine: 50 years in perspective. *Trends Neurosci.* 30 188–193. 10.1016/j.tins.2007.03.002 17368565

[B37] Jang Y. Pletnikova O. Troncoso J. Pantelyat A. Dawson T. Rosenthal L. (2023). Mass spectrometry-based proteomics analysis of human substantia nigra from Parkinson’s disease patients identifies multiple pathways potentially involved in the disease. *Mol. Cell. Proteomics* 22:100452. 10.1016/j.mcpro.2022.100452 36423813 PMC9792365

[B38] Jerber J. Seaton D. Cuomo A. Kumasaka N. Haldane J. Steer J. (2021). Population-scale single-cell RNA-seq profiling across dopaminergic neuron differentiation. *Nat. Genet.* 53 304–312. 10.1038/s41588-021-00801-6 33664506 PMC7610897

[B39] Jones J. Arout C. Luba R. Murugesan D. Madera G. Gorsuch L. (2024). The influence of drug class on reward in substance use disorders. *Pharmacol. Biochem. Behav.* 240:173771. 10.1016/j.pbb.2024.173771 38670466 PMC11162950

[B40] Kamath T. Abdulraouf A. Burris S. Langlieb J. Gazestani V. Nadaf N. (2022). Single-cell genomic profiling of human dopamine neurons identifies a population that selectively degenerates in Parkinson’s disease. *Nat. Neurosci.* 25 588–595. 10.1038/s41593-022-01061-1 35513515 PMC9076534

[B41] Kershberg L. Banerjee A. Kaeser P. (2022). Protein composition of axonal dopamine release sites in the striatum. *Elife* 11:e83018. 10.7554/eLife.83018 36579890 PMC9937654

[B42] Kitaoka Y. Uchihashi T. Kawata S. Nishiura A. Yamamoto T. Hiraoka S. (2025). Role and potential of artificial intelligence in biomarker discovery and development of treatment strategies for amyotrophic lateral sclerosis. *Int. J. Mol. Sci.* 26:4346. 10.3390/ijms26094346 40362582 PMC12072360

[B43] Knab F. Guaitoli G. Jarboui M. von Zweydorf F. Isik F. Klose F. (2024). The cellular and extracellular proteomic signature of human dopaminergic neurons carrying the LRRK2 G2019S mutation. *Front. Neurosci.* 18:1502246. 10.3389/fnins.2024.1502246 39726830 PMC11669673

[B44] Krauskopf J. Eggermont K. Madeiro Da Costa R. F. Bohler S. Hauser D. Caiment F. (2022). Transcriptomics analysis of human iPSC-derived dopaminergic neurons reveals a novel model for sporadic Parkinson’s disease. *Mol. Psychiatry* 27 4355–4367. 10.1038/s41380-022-01663-y 35725899

[B45] Kravitz A. Kreitzer A. (2012). Striatal mechanisms underlying movement, reinforcement, and punishment. *Physiology (Bethesda)* 27 167–177. 10.1152/physiol.00004.2012 22689792 PMC3880226

[B46] Lee B. Kim H. Kim H. Jeong H. Kim B. Lee H. (2020). O-GlcNAcylation regulates dopamine neuron function, survival and degeneration in Parkinson disease. *Brain* 143 3699–3716. 10.1093/brain/awaa320 33300544 PMC7805798

[B47] Li Q. (2010). Advances in protein turnover analysis at the global level and biological insights. *Mass Spectrom. Rev.* 29 717–736. 10.1002/mas.20261 19757418

[B48] Li W. Dasgupta A. Yang K. Wang S. Hemandhar-Kumar N. Chepyala S. (2025). Turnover atlas of proteome and phosphoproteome across mouse tissues and brain regions. *Cell* 188 2267–2287.e21. 10.1016/j.cell.2025.02.021. 40118046 PMC12033170

[B49] Lim L. Egnot C. Papaioannou P. Yip S. (2025). The hypothalamic arcuate nucleus dopaminergic neurons: More than just prolactin secretion. *Endocrinology* 166:bqaf025. 10.1210/endocr/bqaf025 39919032 PMC11837187

[B50] Liss B. Roeper J. (2008). Individual dopamine midbrain neurons: Functional diversity and flexibility in health and disease. *Brain Res. Rev.* 58 314–321. 10.1016/j.brainresrev.2007.10.004 18023878

[B51] Luo S. Wang D. Zhang Z. (2023). Post-translational modification and mitochondrial function in Parkinson’s disease. *Front. Mol. Neurosci.* 16:1329554. 10.3389/fnmol.2023.1329554 38273938 PMC10808367

[B52] Luquet E. Biesemann C. Munier A. Herzog E. (2017). Purification of synaptosome populations using fluorescence-activated synaptosome sorting. *Methods Mol. Biol.* 1538 121–134. 10.1007/978-1-4939-6688-2_10 27943188

[B53] Mandel S. Sagi Y. Amit T. (2007). Rasagiline promotes regeneration of substantia nigra dopaminergic neurons in post-MPTP-induced Parkinsonism via activation of tyrosine kinase receptor signaling pathway. *Neurochem. Res.* 32 1694–1699. 10.1007/s11064-007-9351-8 17701352

[B54] Meyer K. Woodworth M. Goncalves M. Yue M. AlJandal H. Morton S. (2025). Altered protein phosphorylation in a novel midbrain organoid model for bipolar disorder. *bioRxiv* [Preprint] 10.1101/2025.04.01.64664210.1101/2025.04.01.646642

[B55] Michel P. Hirsch E. Hunot S. (2016). Understanding dopaminergic cell death pathways in parkinson disease. *Neuron* 90 675–691. 10.1016/j.neuron.2016.03.038 27196972

[B56] Monzel A. Smits L. Hemmer K. Hachi S. Moreno E. van Wuellen T. (2017). Derivation of human midbrain-specific organoids from neuroepithelial stem cells. *Stem Cell Rep.* 8 1144–1154. 10.1016/j.stemcr.2017.03.010 28416282 PMC5425618

[B57] Morgenstern M. Peikert C. Lübbert P. Suppanz I. Klemm C. Alka O. (2021). Quantitative high-confidence human mitochondrial proteome and its dynamics in cellular context. *Cell. Metab.* 33 2464–2483.e18. 10.1016/j.cmet.2021.11.001. 34800366 PMC8664129

[B58] Moritz C. Mühlhaus T. Tenzer S. Schulenborg T. Friauf E. (2019). Poor transcript-protein correlation in the brain: Negatively correlating gene products reveal neuronal polarity as a potential cause. *J. Neurochem.* 149 582–604. 10.1111/jnc.14664 30664243

[B59] Novak G. Kyriakis D. Grzyb K. Bernini M. Rodius S. Dittmar G. (2022). Single-cell transcriptomics of human iPSC differentiation dynamics reveal a core molecular network of Parkinson’s disease. *Commun. Biol.* 5:49. 10.1038/s42003-021-02973-7 35027645 PMC8758783

[B60] Ong S. Blagoev B. Kratchmarova I. Kristensen D. Steen H. Pandey A. (2002). Stable isotope labeling by amino acids in cell culture, SILAC, as a simple and accurate approach to expression proteomics. *Mol. Cell. Proteomics* 1 376–386. 10.1074/mcp.m200025-mcp200 12118079

[B61] Paget-Blanc V. Pfeffer M. Pronot M. Lapios P. Angelo M. Walle R. (2022). A synaptomic analysis reveals dopamine hub synapses in the mouse striatum. *Nat. Commun.* 13:3102. 10.1038/s41467-022-30776-9 35660742 PMC9166739

[B62] Plum S. Eggers B. Helling S. Stepath M. Theiss C. Leite R. (2020). Proteomic characterization of synaptosomes from human substantia nigra indicates altered mitochondrial translation in Parkinson’s disease. *Cells* 9:2580. 10.3390/cells9122580 33276480 PMC7761546

[B63] Prelaj A. Miskovic V. Zanitti M. Trovo F. Genova C. Viscardi G. (2024). Artificial intelligence for predictive biomarker discovery in immuno-oncology: A systematic review. *Ann. Oncol.* 35 29–65. 10.1016/j.annonc.2023.10.125 37879443

[B64] Puig S. Xue X. Salisbury R. Shelton M. Kim S. Hildebrand M. (2023). Circadian rhythm disruptions associated with opioid use disorder in synaptic proteomes of human dorsolateral prefrontal cortex and nucleus accumbens. *Mol. Psychiatry* 28 4777–4792. 10.1038/s41380-023-02241-6 37674018 PMC10914630

[B65] Rhee H. Zou P. Udeshi N. Martell J. Mootha V. Carr S. (2013). Proteomic mapping of mitochondria in living cells via spatially restricted enzymatic tagging. *Science* 339 1328–1331. 10.1126/science.1230593 23371551 PMC3916822

[B66] Rosenberger F. Thielert M. Mann M. (2023). Making single-cell proteomics biologically relevant. *Nat. Methods* 20 320–323. 10.1038/s41592-023-01771-9 36899157

[B67] Roux K. Kim D. Raida M. Burke B. A. (2012). promiscuous biotin ligase fusion protein identifies proximal and interacting proteins in mammalian cells. *J. Cell. Biol.* 196 801–810. 10.1083/jcb.201112098 22412018 PMC3308701

[B68] Sabatier P. Lechner M. Guzmán U. Beusch C. Zeng X. Wang L. (2025). Global analysis of protein turnover dynamics in single cells. *Cell* 188 2433–2450.e21. 10.1016/j.cell.2025.03.002. 40168994

[B69] Salamone J. Correa M. (2012). The mysterious motivational functions of mesolimbic dopamine. *Neuron* 76 470–485. 10.1016/j.neuron.2012.10.021 23141060 PMC4450094

[B70] Schmidt S. Stautner C. Vu D. Heinz A. Regensburger M. Karayel O. (2023). A reversible state of hypometabolism in a human cellular model of sporadic Parkinson’s disease. *Nat. Commun.* 14:7674. 10.1038/s41467-023-42862-7 37996418 PMC10667251

[B71] Schwanhäusser B. Busse D. Li N. Dittmar G. Schuchhardt J. Wolf J. (2011). Global quantification of mammalian gene expression control. *Nature* 473 337–342. 10.1038/nature10098 21593866

[B72] Sison S. Vermilyea S. Emborg M. Ebert A. (2018). Using patient-derived induced pluripotent stem cells to identify Parkinson’s disease-relevant phenotypes. *Curr. Neurol. Neurosci. Rep.* 18:84. 10.1007/s11910-018-0893-8 30284665 PMC6739862

[B73] Smits L. Schwamborn J. (2020). Midbrain organoids: A new tool to investigate Parkinson’s disease. *Front. Cell. Dev. Biol.* 8:359. 10.3389/fcell.2020.00359 32509785 PMC7248385

[B74] Stauch K. Villeneuve L. Purnell P. Ottemann B. Emanuel K. Fox H. (2016). Loss of Pink1 modulates synaptic mitochondrial bioenergetics in the rat striatum prior to motor symptoms: Concomitant complex I respiratory defects and increased complex II-mediated respiration. *Proteomics Clin. Appl.* 10 1205–1217. 10.1002/prca.201600005 27568932 PMC5810131

[B75] Steen H. Mann M. (2004). The ABC’s (and XYZ’s) of peptide sequencing. *Nat. Rev. Mol. Cell. Biol.* 5 699–711. 10.1038/nrm1468 15340378

[B76] Teng P. Barakat W. Tran S. Tran Z. Bateman N. Conrads K. (2023). Brain proteomic atlas of alcohol use disorder in adult males. *Transl. Psychiatry* 13:318. 10.1038/s41398-023-02605-0 37833300 PMC10575941

[B77] Thompson A. Schäfer J. Kuhn K. Kienle S. Schwarz J. Schmidt G. (2003). Tandem mass tags: A novel quantification strategy for comparative analysis of complex protein mixtures by MS/MS. *Anal. Chem.* 75 1895–1904. 10.1021/ac0262560 12713048

[B78] van Oostrum M. Blok T. Giandomenico S. Tom Dieck S. Tushev G. Fürst N. (2023). The proteomic landscape of synaptic diversity across brain regions and cell types. *Cell* 186 5411–5427.e23. 10.1016/j.cell.2023.09.028. 37918396 PMC10686415

[B79] Vogt Weisenhorn D. Giesert F. Wurst W. (2016). Diversity matters - heterogeneity of dopaminergic neurons in the ventral mesencephalon and its relation to Parkinson’s Disease. *J. Neurochem.* 139 8–26. 10.1111/jnc.13670 27206718 PMC5096020

[B80] Wise R. (2004). Dopamine, learning and motivation. *Nat. Rev. Neurosci.* 5 483–494. 10.1038/nrn1406 15152198

[B81] Wu X. Xu M. Geng M. Chen S. Little P. Xu S. (2023). Targeting protein modifications in metabolic diseases: Molecular mechanisms and targeted therapies. *Signal Transduct. Target Ther.* 8:220. 10.1038/s41392-023-01439-y 37244925 PMC10224996

[B82] Wulf M. Barkovits K. Schork K. Eisenacher M. Riederer P. Gerlach M. (2022). Neuromelanin granules of the substantia nigra: Proteomic profile provides links to tyrosine hydroxylase, stress granules and lysosomes. *J. Neural Transm.* 129 1257–1270. 10.1007/s00702-022-02530-4 35852604 PMC9468065

[B83] Xu Y. Fan X. Hu Y. (2021). In vivo interactome profiling by enzyme-catalyzed proximity labeling. *Cell. Biosci.* 11:27. 10.1186/s13578-021-00542-3 33514425 PMC7847152

[B84] Zaccaria A. Antinori P. Licker V. Kövari E. Lobrinus J. Burkhard P. (2022). Multiomic analyses of dopaminergic neurons isolated from human substantia nigra in Parkinson’s disease: A descriptive and exploratory study. *Cell. Mol. Neurobiol.* 42 2805–2818. 10.1007/s10571-021-01146-8 34528139 PMC9561004

[B85] Zhao H. Chen P. Gao X. Huang Z. Yang P. Shen H. (2025). Spatiotemporal proteomic and transcriptomic landscape of DAT+ dopaminergic neurons development and function. *iScience* 28:112115. 10.1016/j.isci.2025.112115 40201125 PMC11978345

[B86] Zheng X. Mund A. Mann M. (2025). Deciphering functional tumor-immune crosstalk through highly multiplexed imaging and deep visual proteomics. *Mol. Cell.* 85 1008–1023.e7. 10.1016/j.molcel.2024.12.023. 39814024

